# Complete response of extensive cutaneous angiosarcoma of the scalp after PD1 immunotherapy combined with radiotherapy: A report of 2 cases

**DOI:** 10.1016/j.jdcr.2026.08.023

**Published:** 2026-08-22

**Authors:** Anna-Sophia Leven, Christoph Pöttgen, Georg Lodde, Lisa Zimmer, Elisabeth Livingstone, Dirk Schadendorf, Alpaslan Tasdogan, Selma Ugurel

**Affiliations:** a Department of Dermatology, University Hospital Essen & German Cancer Consortium (DKTK), Partner Site Essen, Essen, Germany; b Department of Radiotherapy, West German Cancer Center, University of Duisburg-Essen, University Hospital Essen, Essen, Germany; c German Cancer Consortium, Partner Site Essen, Essen, Germany; d National Center for Tumor Diseases (NCT)-West, Campus Essen, & Research Alliance Ruhr, Research Center One Health, University Duisburg-Essen, Essen, Germany; e Department of Dermatology, Bielefeld University, Medical School and University Medical Center OWL, Klinikum Bielefeld Rosenhöhe, Bielefeld, Germany

**Keywords:** Cutaneous angiosarcoma, Immune checkpoint inhibition, skin cancer, soft tissue sarcoma

## Introduction

Cutaneous angiosarcoma (CAS) is a rare, highly aggressive skin cancer that comprises only 1% to 2% of soft tissue sarcomas and is characterized by early metastasis.^1^^,^^2^ Despite multimodal treatment including surgery and systemic therapy, the prognosis remains poor particularly for tumors of the head and neck region with reported 5-year overall survival rates ranging from 10% to 35%.^3^ The current standard of care includes wide-margin excision, followed by radiation therapy (RT) and/or chemotherapy (CT).^4^ If surgery is not reasonable, definitive RT may be considered. Despite the use of different treatment modalities as CT, RT and kinase inhibitors, treatment outcomes for angiosarcoma remain poor. This is primarily because angiosarcoma is rare and approved ("on label") therapies are only available in exceptional cases. Recently, treatment approaches using immune checkpoint inhibitors (ICI) have been discussed.^5^, ^6^, ^7^, ^8^

The rationale for ICI treatment in CAS is based on the tumor’s high mutational burden (TMB). Significant rates of PD1-expressing T-cells infiltrating the tumor and PD-L1 expression on tumor cells have been described. Honda et al found PD-L1 positivity in 30% of 106 cases and PD1-positive T cell infiltrates in 17% with both associated with a favorable prognosis.^5^ A case series by Florou et al reported an objective response after 12 weeks of pembrolizumab in 5 of 7 patients.^6^ In a prospective multicenter phase II trial (SWOG S1609), the combination of ipilimumab and nivolumab achieved an ORR of 25% among 16 patients with metastatic or unresectable angiosarcoma, with notably higher response rates (60%) observed in patients with primary cutaneous tumors of the scalp or face.^7^ However in the largest prospective trial to date (AngioCheck), nivolumab monotherapy in pretreated CAS patients resulted in a modest ORR of 13%.^8^ This suggests that while some patients benefit, overall response rates remain limited and reliable predictive biomarkers are lacking.

## Case report

Here we present 2 patients with extensive CAS of the scalp who received PD1 ICI in combination with RT. An 84-year-old female with a history of mild psoriasis since childhood (well controlled with topical therapy and never required systemic treatment), heart valve replacement and arterial hypertension. A 92-year-old male with no relevant comorbidities. Initially the tumors presented as red to livid macules on which papules and nodes rapidly developed reaching diameters of up to 20 cm with partial ulceration. Histopathological evaluation of the biopsy confirmed the diagnosis of cutaneous angiosarcoma. Immunohistochemical staining revealed strong expression of CD31 in the tumor infiltrating cells, positivity for D240 and a markedly elevated proliferative index as demonstrated by Ki-67 immunostaining. Whole-body CT/MRI imaging showed a soft-tissue density lesion in the scalp corresponding to the angiosarcoma and revealed mildly prominent mediastinal and bilateral inguinal lymph nodes without pathological configuration and with no evidence of distant metastatic. The cases were discussed in our interdisciplinary tumor board. Surgery was not considered as reasonable and combination therapy with RT and PD1 ICI immunotherapy was recommended.

**Fig 1:**
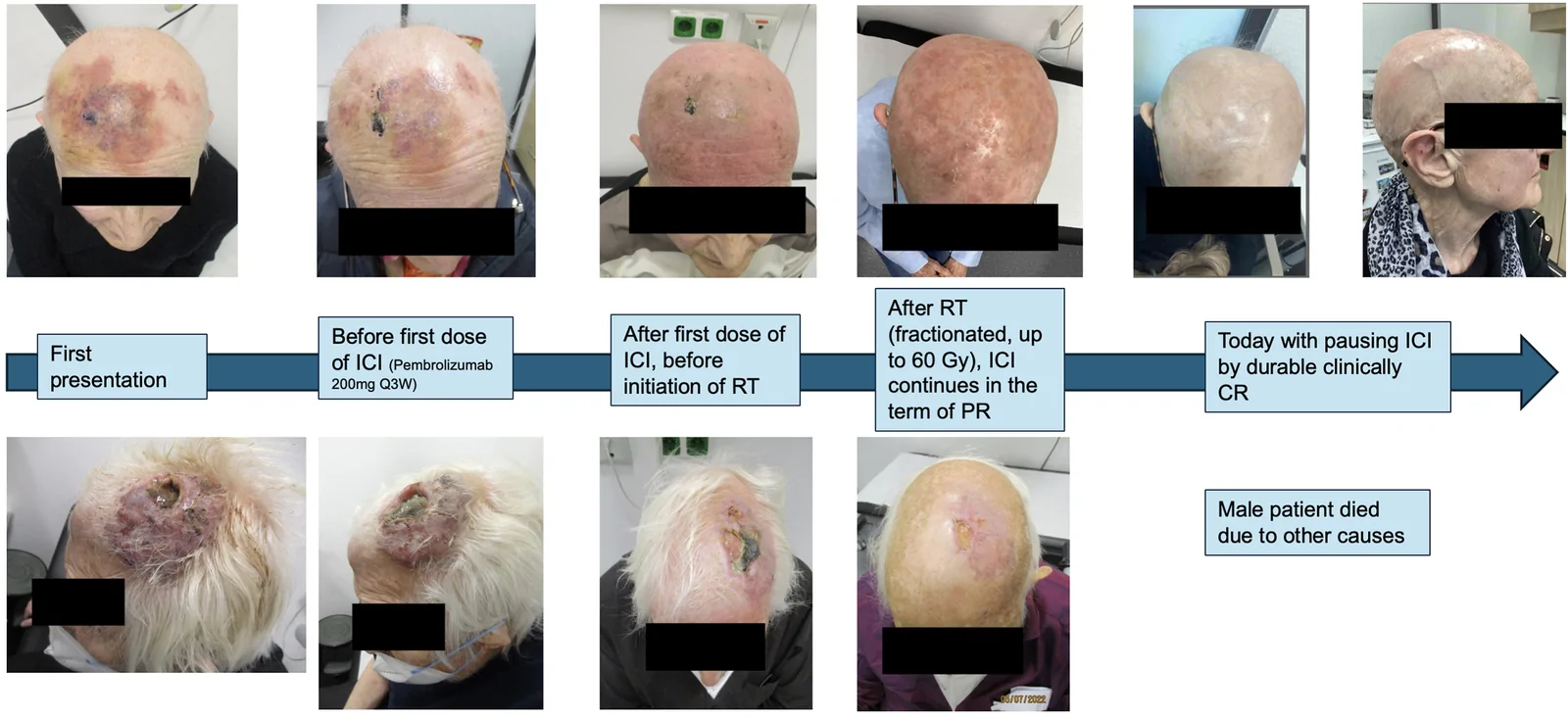
Overview of the treatment regimen and clinical progress of the two patients (female above and male below) (*PR* = Partial response; *CR* = complete response; *ICI* = immune checkpoint inhibition; *RT* = radiotherapy). Leven AS et al: Complete response after PD1-immune checkpoint inhibition therapy in combination with radiotherapy in cutaneous angiosarcoma.

Both promptly started ICI with pembrolizumab (200 mg fixed dose every 3 weeks [Q3W]). After the first dose RT was initiated. Fractionated radiation was performed with 2 Gy up to a total of 60 Gy. MRI and CT scans after 3 months showed a partial remission. Pembrolizumab dosing was extended to 400 mg fixed dose every 6 weeks (Q6W) in the male and 2 months later also in the female patient. Both patients tolerated ICI and radiation therapy well with no treatment-related adverse events observed. A detailed time course is provided in Figure 1.

After completion of RT, ICI therapy was continued. Upon achieving a clinical complete response (CR) after 9 doses of pembrolizumab, the female patient was recommended to pause treatment by interdisciplinary tumor board decision. The patient currently still is in CR 21 months after start of pembrolizumab. The male patient also achieved a CR, but died of a stroke at 8 months after start of pembrolizumab (probably unrelated to pembrolizumab).

## Discussion

The favorable response to ICI can be explained by the biological characteristics of angiosarcomas in the head and neck region. Recent studies have shown that angiosarcomas are biologically heterogeneous with significant differences depending on their anatomical site of origin (Table I). Scalp and face angiosarcomas are characterized by a high tumor mutational burden (TMB), frequent TP53 and POT1 mutations and a UV-induced mutational signature (SBS7a/7b). These tumors also display a highly inflamed immune microenvironment with increased immune cell infiltration and PD-L1 expression, which may contribute to their better response to ICI.^9^ In line with these findings, both of our cases demonstrated PD-L1 expression by immunohistochemistry with more than 5% of tumor cells staining positive.

**Table I:** Molecular and genetic differences between angiosarcoma subtypes by anatomic site^9^; UV (ultraviolet radiation); IR (ionising radiation); TMB (tumor mutational burden)

Site	Key molecular features	Mutational signature	Immune microenvironment	Clinical notes
Head/neck/scalp	TP53, POT1, high TMB	UV	Highly inflamed, PD-L1+	Best ICI response, aggressive
Breast (secondary)	MYC amplification, DNA repair genes	IR	Variable	Often post-radiation, distinct genes
Extremities/trunk	Angiogenesis/cell cycle genes	No UV/IR signature	Less inflamed	Deep soft tissue, aggressive course

RT can also contribute to favorable therapeutic outcomes. Its benefit in head and neck angiosarcoma is well established. In patients which are not eligible for surgery definitive radiotherapy is often indicated. Given the aggressive biology of CAS and the high risk of recurrence, multimodal management is generally recommended.^10^

To our knowledge these are the first 2 reports of CAS in which combined RT and PD-1 ICI therapy achieved a CR including a durable long-term response in 1 patient. These cases suggest that PD-1 blockade may be particularly effective when administered with RT in this highly aggressive disease. A potential mechanistic explanation is that RT induces DNA damage and immunogenic cell death, which may increase tumour susceptibility and enhance tumor immunogenicity, thereby augmenting responses to ICI.

These cases suggest that PD-1 immunotherapy combined with RT may represent a promising treatment option for CAS that is not amenable to surgery.

## Conflicts of interest

Ugurel declares research support from Bristol Myers Squibb and Merck; speakers and advisory board honoraria from Merck Sharp & Dohme, Merck, and Menarini Stemline; and meeting and travel support from Bristol-Myers Squibb, Merck Sharp & Dohme, Novartis, Pierre Fabre, Regeneron and Sun Pharma; outside the submitted work. Leven, Pöttgen, Lodde, Zimmer, Livingstone, Schadendorf, and Tasdogan have declared no conflicts of interest.
